# Classical scrapie prions in ovine blood are associated with B lymphocytes and platelet-rich plasma

**DOI:** 10.1186/1746-6148-7-75

**Published:** 2011-11-23

**Authors:** Rohana P Dassanayake, David A Schneider, Thomas C Truscott, Alan J Young, Dongyue Zhuang, Katherine I O'Rourke

**Affiliations:** 1 Department of Veterinary Microbiology and Pathology, College of Veterinary Medicine, Washington State University, Pullman, WA 99164-7040 USA; 2 Animal Disease Research Unit, Agricultural Research Service, U.S. Department of Agriculture, Pullman, WA 99164-6630 USA; 3 Department of Veterinary and Biomedical Science, South Dakota State University, Brookings, SD 57007, USA

## Abstract

**Background:**

Classical scrapie is a naturally occurring transmissible spongiform encephalopathy of sheep and goats characterized by cellular accumulation of abnormal isoforms of prion protein (PrP^Sc^) in the central nervous system and the follicles of peripheral lymphoid tissues. Previous studies have shown that the whole blood and buffy coat blood fraction of scrapie infected sheep harbor prion infectivity. Although PrP^Sc ^has been detected in peripheral blood mononuclear cells (PBMCs), plasma, and more recently within a subpopulation of B lymphocytes, the infectivity status of these cells and plasma in sheep remains unknown. Therefore, the objective of this study was to determine whether circulating PBMCs, B lymphocytes and platelets from classical scrapie infected sheep harbor prion infectivity using a sheep bioassay.

**Results:**

Serial rectal mucosal biopsy and immunohistochemistry were used to detect preclinical infection in lambs transfused with whole blood or blood cell fractions from preclinical or clinical scrapie infected sheep. PrP^Sc ^immunolabeling was detected in antemortem rectal and postmortem lymphoid tissues from recipient lambs receiving PBMCs (15/15), CD72^+ ^B lymphocytes (3/3), CD21^+ ^B lymphocytes (3/3) or platelet-rich plasma (2/3) fractions. As expected, whole blood (11/13) and buffy coat (5/5) recipients showed positive PrP^Sc ^labeling in lymphoid follicles. However, at 549 days post-transfusion, PrP^Sc ^was not detected in rectal or other lymphoid tissues in three sheep receiving platelet-poor plasma fraction.

**Conclusions:**

Prion infectivity was detected in circulating PBMCs, CD72^+ ^pan B lymphocytes, the CD21^+ ^subpopulation of B lymphocytes and platelet-rich plasma of classical scrapie infected sheep using a sheep bioassay. Combining platelets with B lymphocytes might enhance PrP^Sc ^detection levels in blood samples.

## Background

Prion diseases or transmissible spongiform encephalopathies (TSEs) are unique, fatal neurodegenerative disorders that affect a variety of species including sheep and goats (scrapie), cattle (bovine spongiform encephalopathy, BSE), human (Creutzfeldt-Jakob disease, CJD), mink (transmissible mink encephalopathy, TME), deer, elk and moose (chronic wasting disease, CWD). A characteristic feature of TSEs is the accumulation of an alternative conformational isoform (PrP^Sc^) of the host-encoded normal prion protein (PrP^c^) in the central nervous system [1,2]. In classical ovine scrapie, deposition of PrP^Sc ^in the lymphoreticular system precedes accumulation in the central nervous system [3]. PrP^Sc ^is readily detected in this preclinical stage by biopsy of the lymphoid tissue in the nictitating membrane [4,5] or in the rectoanal mucosa-associated lymphoid tissue (RAMALT; [6,7]).

The early and persistent presence of PrP^Sc ^in the lymph nodes in classical ovine scrapie, human variant CJD (vCJD), CWD and most rodent scrapie models suggests that prions are disseminated in the peripheral blood and/or lymphatics and that peripheral blood might be a suitable target for preclinical diagnostic testing. Prion infectivity in ovine blood was confirmed by transfusions of whole blood, buffy coat, red cell concentrates, plasma or platelets from donor sheep with experimental BSE or classical scrapie [8-11]. PrP^Sc ^was detected in PBMCs of 10 of 10 clinical scrapie infected sheep using protein misfolding cyclic amplification (PMCA) [12] but in only 44 of 80 clinical scrapie infected sheep when using a conventional enzyme linked immunosorbent assay (ELISA) [13]. A recent ELISA-based study concluded that PrP^Sc ^was principally associated with a subpopulation of B lymphocytes in scrapie infected sheep [14]. Although PrP^Sc ^was detected in B lymphocytes in 11 of 11 clinical scrapie infected sheep, B lymphocytes from only three of five scrapie infected sheep at the preclinical stage were positive for PrP^Sc ^by ELISA. PrP^Sc ^has also been detected in plasma samples collected from both preclinical and clinical scrapie infected sheep but only after combining a novel surround optical fiber immunoassay (SOFIA) with limited PrP^Sc ^amplification by PMCA [15].

Although PrP^Sc ^has been detected in the blood of scrapie-infected sheep by enriching conventional ELISA-based assay with cellular fractions including PBMCs or B lymphocytes, the frequency of such detection was lower during the preclinical stage of the disease. Thus, the objectives of this study were to identify blood fractions of sheep which harbor relatively high levels of prion infectivity, including during the preclinical stage of disease. The present study used a short-observation transfusion model in the natural host to determine if relatively high infectivity was present in the total PBMC population, a CD72^+ ^pan B lymphocyte population, a CD21^+ ^subpopulation of B lymphocytes and either platelet-rich or platelet-poor plasma isolated from the blood of classical scrapie-infected sheep. In addition, it was also determined if high infectivity could be demonstrated in both platelet-rich and platelet-poor plasma during clinical disease. Such demonstrations should help guide further efforts toward improvement of ELISA-based scrapie detection sensitivity by pre-assay enrichment with relevant blood fractions.

## Results

### Scrapie infectivity associates with blood components

#### (i) Whole blood transfused recipients develop preclinical scrapie

**Figure 1:**
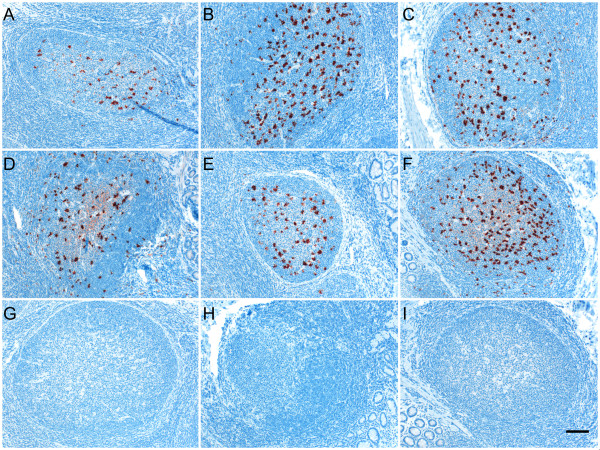
**Immunolabeling of PrP^Sc ^in the rectoanal mucosa-associated lymphoid tissues of recipient sheep transfused with different blood components**. PrP^Sc ^immunolabeling (dark red) was visible in the RAMALT follicles of recipient sheep receiving either whole blood (A), buffy coat (B), PBMCs (C), CD72^+ ^B lymphocytes (D), CD21^+ ^B lymphocytes (E) or platelet-rich plasma (F) fractions but not after receiving a platelet-poor plasma (G) fraction when labeled with anti-prion mAbs. PrP^Sc ^immunolabeling was not observed in RAMALT follicles of scrapie negative (H) sheep or when using an isotype-matched control mAb (I; same tissue block shown in F). Scale bar = 50 μm.

Whole blood was collected from three preclinical and three clinical scrapie infected donor sheep and different blood volumes were transfused to 13 recipients as shown in Table 1. Three of four PRNP MARQ/MVRQ recipients and all four MARQ/MARQ recipients became antemortem RAMALT positive for PrP^Sc ^between 123 to 235 days post transfusion (dpt) and 222 to 252 dpt, respectively (Table 1). None of these eight animals showed any signs of scrapie when they were euthanized at 236 to 267 dpt. IHC analysis of postmortem rectal and retropharyngeal lymph nodes of all eight animals showed positive PrP^Sc ^labeling in lymphoid follicles (Table 2 Figure 1A). PrP^Sc ^was undetectable in antemortem and postmortem rectal tissues and other lymphoid tissues of two MARQ/TARQ recipients (Tables 1 and 2). PrP^Sc ^labeling was also not detected in antemortem rectal biopsies of all three TARQ/MVRQ animals; however when necropsied rectal tissues of two animals and retropharyngeal lymph nodes of all three animals showed positive labeling for PrP^Sc ^(Table 2). Clinical signs were not observed in any of these three recipients and PrP^Sc ^was undetectable in brain tissues of all the animals.

**Table 1:** Sheep transfused with different blood components from scrapie infected sheep.

Transfusion types					Transfusion outcomes				
**Blood component **	**blood volume (mL)**^1^	**Donor ID**	**Clinical status **	**Donor *PRNP***	**Recipient *PRNP***	**Recipient ID**	**Rectal tissues PrP^Sc ^detection by IHC**	**dpt to first positive biopsy**	**dpt at necropsy**

Whole blood	50	3806/3807	Preclinical	MVRQ/MVRQ	MARQ/MVRQ	4128/4132/4134	Positive	156-235	267
	
	131	3178	Preclinical	MARQ/MARQ	MARQ/MARQ	3668	Positive	252	261
					
					MARQ/TARQ	3669/3670	Not detected		236-261
	
	135	3187	Clinical	MARQ/MVRQ	TARQ/MVRQ	3613/3622	Positive		185-213
		3188	Clinical	MARQ/MVRQ	TARQ/MVRQ	3628	Not detected		213
					
					MARQ/MVRQ	3672	Positive	123	215
	
	119-131	3199	Clinical	MARQ/MARQ	MARQ/MARQ	3665/3666/3667	Positive	222-252	236-261

Buffy coat	100	3345	Clinical	MARQ/MARQ	MARQ/MARQ	3823/3833	Positive	239-288	275-306
					
					MARQ/MARQ	3832	Positive	549	1259
					
					MARQ/TARQ	3819	Positive	757	1391
					
					MARQ/TARQ	3825	Not detected		1391

PBMCs	50	4073	Preclinical	MVRQ/MVRQ	MVRQ/MVRQ	4302	Positive	182	646
					
					MARQ/MVRQ	4288/4309	Positive	182	646
					
					MARQ/MVRQ	4304/4305	Positive		251
	
	100	3806/3807	Preclinical	MVRQ/MVRQ	MARQ/MVRQ	4118/4119/4123	Positive	153-214	306
					
					TARQ/MVRQ	4120	Positive	153	306
	
	116	3774	Preclinical	MVRQ/MVRQ	MVRQ/MVRQ	3824	Positive	175	183
					
					MARQ/MVRQ	3921	Positive	175	183
					
					MARQ/MVRQ	3920	Positive		187
	
	50^@^	4125	Preclinical	MVRQ/MVRQ	MVRQ/MVRQ	4378/4380	Positive	125	181
					
					MVRQ/MVRQ	4379	Positive		181

CD72^+ ^B cells	50^#^	4125	Preclinical	MVRQ/MVRQ	MVRQ/MVRQ	4384/4385/4386	Positive	125-152	181

CD21^+ ^B cells	50^&^	4125	Preclinical	MVRQ/MVRQ	MVRQ/MVRQ	4381/4383	Positive	125-152	187
					
					MVRQ/MVRQ	4382	Positive		187

Platelet-rich	180	3199	Clinical	MARQ/MARQ	MARQ/MARQ	3606	Positive	218	357

plasma	100	3345	Clinical	MARQ/MARQ	MARQ/MARQ	3820	Positive	288	554
					
					MARQ/MARQ	3821	Not detected		552

Platelet-poor plasma	100	3345	Clinical	MARQ/MARQ	MARQ/MARQ	3822/3836/3838	Not detected		549

abbreviations: PBMCs = peripheral blood mononuclear cells; IHC = immunohistochemistry on antemortem and postmortem rectal tissues; *PRNP *= haplotypes shown include deduced amino acids at codons 112, 136, 154, 171; dpt = days post-transfusion; cells labeled with magnetic beads: ^@^CD18-labeled PBMCs; ^#^CD72-labeled B cells (Pan B lymphocytes); ^&^CD21-labeled CD21^+ ^B cells (a subpopulation of B lymphocytes), these cells were labeled with primary and magnetic microbeads coupled secondary antibodies

^1^Total whole blood volume from which each inoculum was derived per recipient animal.

**Table 2:** Summary of PrP^Sc ^detection in multiple tissues from sheep transfused with different blood components from scrapie infected sheep.

Blood components	Recipient	PrP^Sc ^detection by IHC			*Scrapie
			
	*PRNP*	Antemortem	Postmortem		positive (%)
		
		Rectal tissues	Rectal tissues	Other lymphoid tissues	
Whole blood	MARQ/MARQ	4/4	4/4	4/4	100
	
	MARQ/MVRQ	3/4	4/4	4/4	100
	
	MARQ/TARQ	0/2	0/2	0/2	0
	
	TARQ/MVRQ	0/3	2/3	3/3	100

Buffy coat	MARQ/MARQ	3/3	3/3	3/3	100
	
	MARQ/TARQ	1/2	1/2	2/2	100

PBMCs	MVRQ/MVRQ	4/5	5/5	5/5	100
	
	MARQ/MVRQ	6/9	7/9	9/9	100
	
	TARQ/MVRQ	1/1	1/1	1/1	100

CD72^+ ^B lymphocytes	MVRQ/MVRQ	3/3	3/3	3/3	100

CD21^+ ^B lymphocytes	MVRQ/MVRQ	2/3	3/3	3/3	100

Platelet-rich plasma	MARQ/MARQ	2/3	2/3	2/3	67

Platelet-poor plasma	MARQ/MARQ	0/3	0/3	0/3	0

Other lymphoid tissues = retropharyngeal lymph nodes, palatine tonsil, spleen, ileo-cecal junction, ileo-cecal lymph node or mesenteric lymph nodes; IHC = immunohistochemistry; *Scrapie positive PrP^Sc ^was detected in one or more lymphoid tissues at necropsy

#### (ii) Buffy coat transfused recipients develop preclinical scrapie

Buffy coat fraction was prepared from one clinical scrapie infected donor sheep and transfused to five recipients (Table 1). All three MARQ/MARQ buffy coat recipients became antemortem RAMALT biopsy positive for PrP^Sc ^between 239 to 549 dpt (Table 1 Figure 1B). Postmortem rectal tissues and retropharyngeal lymph nodes along with other lymphoid tissues of these three animals showed positive PrP^Sc ^labeling in lymphoid follicles (Table 2) although PrP^Sc ^was not detected in the brain tissues. Two of these three animals did not show clinical signs of scrapie when they were euthanized at 275 and 306 dpt. Clinical signs of scrapie developed in the remaining animal, which was euthanized at 1259 dpt. Brain tissue of this animal (ID 3832) was positive for PrP^Sc ^by both IHC and WB assays. One of two remaining buffy coat recipients with the MARQ/TARQ PRNP genotype became rectal biopsy positive for PrP^Sc ^labeling in lymphoid follicles at 757 dpt while PrP^Sc ^labeling was not detected from the remaining other animal at 1095 dpt. There were no clinical signs of scrapie in either of these two animals when euthanized at 1391 dpt (Table 1). Postmortem rectal tissues and retropharyngeal lymph nodes along with other lymphoid tissues of the RAMALT positive animal (ID 3819) showed positive PrP^Sc ^labeling in lymphoid follicles and the brain tissues by IHC (Table 2). Lymphoid follicles of retropharyngeal lymph nodes and ileocecal junction of the other animals (ID 3825) showed positive PrP^Sc ^labeling (Table 2).

#### (iii) Peripheral blood mononuclear cell transfused recipients develop preclinical scrapie

PBMCs were prepared from five preclinical scrapie infected donor sheep and transfused to 15 recipients (Table 1). Antemortem rectal biopsies from four of five MVRQ/MVRQ recipients (including three CD18^+^-labeled PBMC recipients) became positive for PrP^Sc ^labeling between 125 and 182 dpt (Table 1 Figure 1C). There were no clinical signs of scrapie in any of these animals. All five were euthanized between 181 and 646 dpt. Rectal tissues, retropharyngeal lymph nodes and most other lymphoid tissues of all five animals showed positive PrP^Sc ^labeling in lymphoid follicles (Tables 1 and 2). PrP^Sc ^labeling in brain tissues was only detected in one animal (ID 4302). Antemortem rectal biopsies of six of nine MARQ/MVRQ animals showed positive PrP^Sc ^labeling in lymphoid follicles between 153 and 214 dpt. Rectal tissues collected at necropsy from two of three previously RAMALT PrP^Sc ^undetectable animals remained undetectable for PrP^Sc ^labeling in lymphoid follicles (Table 2). Only the retropharyngeal lymph node of one recipient (ID 4305) showed positive PrP^Sc ^labeling while most of the other lymphoid tissues collected at necropsy from all the other eight animals showed positive PrP^Sc ^labeling in lymphoid follicles. Brain tissues of two animals necropsied at 646 dpt showed positive PrP^Sc ^labeling while PrP^Sc ^labeling was not detected in all the other animals. Antemortem rectal tissue of the remaining TARQ/MVRQ recipient was positive for PrP^Sc ^at 153 dpt. Rectal tissues, retropharyngeal lymph nodes and several other lymphoid tissues collected at necropsy from this animal showed positive PrP^Sc ^labeling in lymphoid follicles (Table 2). However, PrP^Sc ^was not undetected in the brain tissues.

#### (iv) B lymphocyte recipients develop preclinical scrapie

Since PBMCs from four donor animals were able to cause scrapie in recipient sheep, another preclinical scrapie infected sheep was selected as a blood donor to identify prion infectivity in B lymphocytes. As a control for the study, three recipients were transfused with CD18-labeled PBMCs and all the recipients in this group showed positive PrP^Sc ^labeling in lymphoid follicles (Table 1). Since MVRQ/MVRQ animals became PrP^Sc ^positive more quickly than other genotypes, six lambs with this genotype were selected for the B lymphocyte transfusion experiment. CD72^+ ^B lymphocytes and CD21^+ ^B lymphocytes were separated from PBMCs using a magnetic labeling procedure with 95% purity as assessed by flow cytometry (data not shown). IHC examination of antemortem rectal biopsies collected from all three CD72^+ ^(Figure 1D) and two of three CD21^+ ^(Figure 1E) B lymphocyte recipients showed positive PrP^Sc ^labeling between 125 and 152 dpt (Table 1). Rectal as well as all the other lymphoid tissues collected from both types of B lymphocyte recipients at necropsy showed positive PrP^Sc ^labeling in lymphoid follicles (Table 2). However, PrP^Sc ^was not detected from any of the brain tissues of all six animals.

#### (v) Platelet-rich plasma recipients develop preclinical scrapie

Platelet-rich and platelet-poor plasma was prepared from two clinical scrapie infected donor sheep and transfused to six recipients. Three MARQ/MARQ lambs were transfused with platelet-rich plasma prepared from two clinical scrapie donors (Table 1). Antemortem rectal biopsies of two of three recipients showed positive PrP^Sc ^labeling between 218 and 288 dpt (Figure 1F). The three animals did not show any clinical signs of scrapie when they were euthanized at 357 or 554 dpt. PrP^Sc ^was detected in retropharyngeal lymph nodes and all the other lymphoid tissues examined in the two rectal biopsy positive animals, but PrP^Sc ^was not detected in the same tissues in the previously biopsy negative animal (Table 2). In addition, PrP^Sc ^remained undetectable in brain tissues from these animals. PrP^Sc ^was not detected in the tissues from the three recipients of platelet-poor plasma when euthanized at 549 dpt (Table 1 Table 2 Figure 1G).

## Discussion

Removal of exogenous and endogenous prion infectivity from red blood cell preparations of scrapie-infected hamster blood by leukoreduction filters resulted in significant reduction of scrapie infection in hamsters following transfusion [16]. Leukodepletion significantly reduced the risk of vCJD transmission in human following blood transfusion recipients as well [17,18]. However, a recent study by McCutcheon et al., (2011) revealed that leucoreduction did not prevent the BSE transmission to sheep following a single blood transfusion [11]. Detection of PrP^Sc ^labeling in the lymphoid tissues and development of clinical scrapie in whole blood and buffy coat transfusion recipients in this study as well as in previous studies [8-10] confirmed that prion infectivity is associated with blood from classical scrapie infected sheep. Although previous sheep blood transfusion studies used a relatively large volume of whole blood (400 - 500 mL), volumes of 50 - 135 mL whole blood from scrapie infected sheep were sufficient to transmit scrapie infection to the most recipients in this study. It is difficult to avoid the loss of PBMCs during density-gradient cell separation and MACS-based cell enrichment procedures (CD72^+ ^and/or CD21^+ ^B lymphocytes) due to the multiple washing steps involved and the inherent limitation to the cell-binding capacity of the columns. Therefore, it is possible that even much lower volumes of scrapie infected sheep blood might be sufficient to cause infection in sheep. Demonstration of prion infectivity in much smaller volumes of blood may be helpful in development of conventional blood-based diagnostic testing for scrapie in sheep.

We used standard IHC detection of PrP^Sc ^in tissues as a surrogate marker for transmission of infectivity to preclinical recipient sheep. Analysis of antemortem and postmortem lymphoid tissues in sheep receiving transfusion with the PBMC fractions were confirmed positive for PrP^Sc ^immunolabeling indicating that prion infectivity is associated with PBMC fraction of sheep blood. Previous studies showed PrP^Sc ^was detected from PBMCs [12,13] and a subpopulation of B lymphocytes [14]. CD72 has been identified as a pan B lymphocyte marker in sheep [19] and also in mice [20]. Approximately 50% of adult sheep B lymphocytes are positive for CD21 [19]. A significant proportion of peripheral lymphocytes recirculate continuously between the blood, the lymph and the tissues. The lymph nodes are the major site of exchange for recirculating lymphocytes between the blood and the lymph. The migration competent or recirculating B lymphocytes readily migrate into the lymphatic recirculation pathway due to the cell surface expression of CD21 and CD62L or L-selectin [19]. Therefore, we used anti-CD72 and anti-CD21 mAbs to isolate pan B lymphocytes and recirculating B lymphocytes from the PBMCs fraction, respectively. Transfusion of CD72^+ ^B lymphocytes or CD21^+ ^B lymphocytes from scrapie infected sheep resulted in PrP^Sc ^detection in lymphoid tissues of recipients. These results are consistent with PrP^Sc ^being detected in MHC class II DQ^+^, sIgM^+^, CD11b^+^, CD11c^+ ^and CD21^+/- ^B lymphocytes in sheep [14], and CD72^+ ^B lymphocytes harboring infectious CWD prions in white-tailed deer [21].

The present study utilized a short observation period in the natural host as a model biased for quick detection of blood fractions having relatively high prion levels. The platelet-poor plasma fraction from scrapie infected sheep did not contain adequate levels of prion infectivity for detection in this model. Two of the three MARQ/MARQ lambs transfused with platelet rich plasma were positive by rectal biopsy 218 and 288 post-transfusion, approximately the same interval seen in MARQ/MARQ lambs transfused with whole blood (222-252 dpt, n = 4) or buffy coat cells (239-288 days, n = 2). CWD and BSE infectivity were associated with platelets or platelet-rich plasma pellets in white-tailed deer [21] and sheep [11], respectively. However, hamsters inoculated with platelets from scrapie infected hamsters did not develop scrapie [22]. The possibility exists that rodent models and ruminants have slightly different cells that contain infectivity or the processing of platelets could affect scrapie infectivity. Other studies have suggested that there is cell-free PrP^Sc ^in sheep plasma [15,22]. Passage of radioactively labeled, highly purified murine PrP^Sc ^through the mouse blood-brain barrier [23] and detection of PrP^Sc ^in sheep circumventricular organs that lack a blood-brain barrier [24] suggest the possibility of cell-free PrP^Sc ^in the blood. Detection of PrP^Sc ^by PMCA in hamster plasma samples devoid of platelets suggests the possibility of cell-free PrP^Sc ^in hamster plasma [25,26]. Similarly, vCJD infectivity in plasma components has been reported from human donors suggesting cell-free plasma can also carry infectious vCJD prions [27,28]. Given the similarity in pathogenesis between BSE and scrapie infection in sheep, one would expect similar outcome from both forms. However, a recent study by McCutcheon et al., (2011) with BSE-infected sheep revealed that platelet-poor plasma supernatant can also efficiently transmit prion infectivity to recipient lambs following a single blood transfusion [11]. The detection of preclinical scrapie in recipient lambs took 594 to 1089 dpt. The lack of PrP^Sc ^detection in lymphoid tissues of platelet-poor plasma recipients in our study could have been due to a lower prion titer in platelet-poor plasma samples and/or necropsy of animals at 550 dpt. Poisson distribution of particles in solution indicates a far greater number of recipient animals per group would have been necessary to distinguish sample fraction "titers". In contrast to sheep, hamster and human findings, recent studies in white-tailed deer and cervidized transgenic mouse revealed that CWD infectivity was not associated with platelet-poor plasma, but with platelets [21]. Although the presence of PrP^Sc ^in hamsters' plasma fraction is confirmed, prion infectivity in that fraction has not yet been reported.

Our previous study demonstrated that one nonsynonymous allele (T112) was associated with prolonged survival in MARQ/TARQ scrapie-exposed sheep compared to MARQ/MARQ sheep [29]. Although the focus of this study was to identify which blood components carry prion infectivity, four MARQ/TARQ and four TARQ/MVRQ animals were included to assess whether T112 polymorphism might delay PrP^Sc ^detection in RAMALT follicles following infection by transfusion. Lack of PrP^Sc ^labeling in RAMALT follicles of six of eight recipients confirms that T112 polymorphism delayed prion accumulation in sheep rectal lymphoid tissues. Although the number of animals used in this study was limited, the delay in PrP^Sc ^accumulation in RAMALT particularly in MARQ/TARQ animals needs to be considered when scrapie diagnosis is determined.

## Conclusions

This study demonstrated that prion infectivity is associated with B lymphocytes and platelet-rich plasma of sheep with classical scrapie. Enrichment of platelets as well as B lymphocytes should enhance assay detection sensitivity for scrapie.

## Methods

### Blood donor and recipient sheep

**Figure 2:**
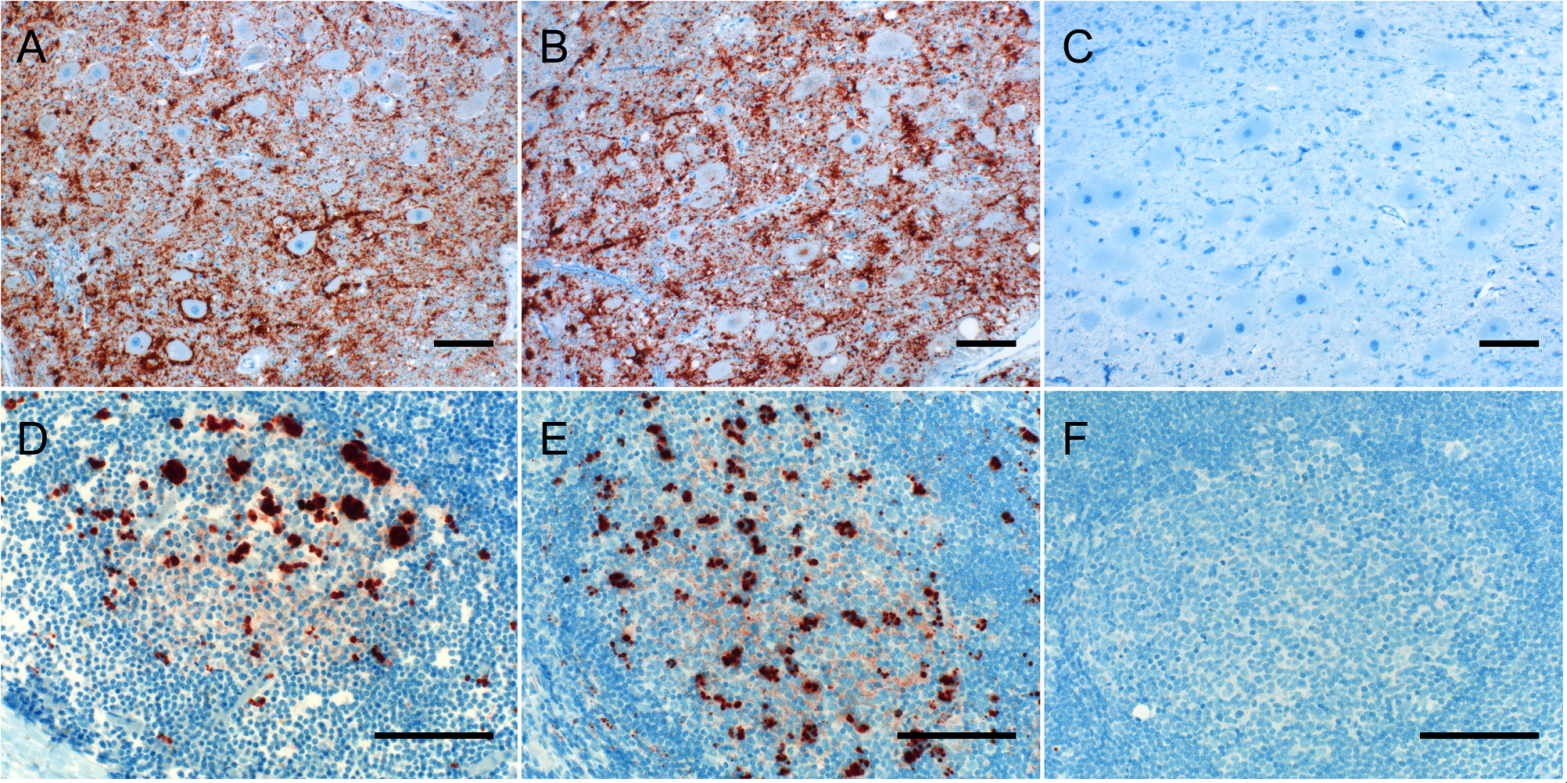
**Immunolabeling of PrP^Sc ^in the brains and retropharyngeal lymph nodes of scrapie infected donor sheep**. Widespread immunolabeling of PrP^Sc ^(dark red) deposits was observed in the brains (A, B) and retropharyngeal lymph node follicles (D, E) of preclinical and clinical scrapie infected donor animals after labeling tissues with anti-prion mAbs, respectively. No PrP^Sc ^immunolabeling was observed in the brain from a sheep without scrapie labeled with the same anti-prion mAbs (C). Immunolabeling of retropharyngeal lymph nodes section from the same scrapie donor sheep (E) block labeled with isotype-matched control mAb (F) shows no non-specific labeling. Scale bars = 50 μm.

All experimental protocols used in this study were approved by the Institutional Animal Care and Use Committee (IACUC) at Washington State University before onset of the study. All donor animals were naturally infected with scrapie and housed at a USDA ARS quarantine facility. Six preclinical (1 MARQ/MARQ, 5 MVRQ/MVRQ) and four clinical (2 MARQ/MARQ, 2 MARQ/MVRQ) scrapie animals were selected as blood donors. Eight of the donor sheep were born and raised in a persistently scrapie-infected flock at the USDA animal research unit at Pullman, WA and developed either preclinical or clinical scrapie. Donor 3774 was received from a privately owned scrapie-infected flock while 4125 was brought from a clean flock and developed preclinical scrapie at our research facility. These animals were mixed breeds of white face or black face sheep. The ages of the donor animals were in the range of 14 to 36 months at the time of blood collection. PrP^Sc ^was detected by IHC in brain and lymphoid tissues collected at necropsy from all the donor sheep (Figure 2). To further confirm PrP^Sc ^detection by IHC, two preclinical and four clinical donor brain tissues were selected for western blot study. Three PK-resistant bands corresponding to di-, mono- and un-glycosylated isoforms of PrP^Sc ^were detected with anti-prion mAb F99/97.6.1 [5] (data not shown). Serum samples collected from donor and recipient animals were negative for ovine progressive pneumonia virus antibody using a cELISA (data not shown). The PRNP genotypes of donor and recipient sheep were determined by sequencing of open reading frame of the PRNP gene [30]. Genotypes are shown by the deduced amino acid residues at positions 112, 136, 154 and 171 respectively. Sheep with PRNP genotypes encoding methionine (M) or threonine (T) at codon 112, alanine (A) or valine (V) at 136, arginine (R) at 154 and glutamine (Q) at 171 were selected for this study. PRNP genotypes and scrapie status of donor and recipient sheep are shown in Table 1. At approximately four-months of age, recipient lambs received from a scrapie negative flock at our facility, USDA ARS sheep experiment station at Dubois, ID, University of Idaho, Moscow, ID or privately owned farms were transferred to isolation buildings or outdoor pens with no direct contact with scrapie infected sheep. These lambs were mixed breed of white face or black face sheep.

### Isolation of blood components and transfusion

All the donor animals were physically restrained during the blood collection and no chemical sedatives were used. During blood transfusion, lambs were also physically restrained and blood or blood components were administered through jugular vein. Jugular venous blood samples from six preclinical and four clinical scrapie positive donor sheep were collected into evacuated containers (Baxter Healthcare Co., Deerfield, IL; 150 - 500 mL capacity) containing acid citrate dextrose as an anticoagulant. The volume of whole blood and the blood fractions derived from the starting volume of donor blood used for transfusion are shown in Table 1. Each animal received the indicated volume of blood or the cells isolated from the indicated volume of whole blood. Plasma from whole blood was initially separated by centrifugation (380 × g for 30 min) and was further centrifuged (1500 × g twice for 15 min each) to prepare platelet-poor plasma. Buffy coat cells collected from centrifuged blood samples were washed twice with phosphate-buffered saline (PBS, Cellgro Inc., Manassas, VA) containing 2 mM EDTA (PBS-EDTA, pH 7.2). Erythrocytes were removed by a short incubation of the buffy coat in erythrocyte lysis solution (Qiagen Inc., Valence, CA) followed by two washes in PBS-EDTA. Thereafter, each buffy coat pellet was re-suspended in 10 mL normal saline and transfused into recipient lambs through the jugular vein. To separate peripheral blood mononuclear cells (PBMCs) from polymorphonuclear cells, buffy coat suspensions were carefully layered onto Accu-Paque™ (Accurate Chemicals, Westbury, NY) density gradient solution (density - 1.088 g/mL; osmolarity - 350 mOsm) and centrifuged (380 × g for 30 min). PBMCs were collected from the plasma-Accu-Paque interface and any remaining erythrocytes were removed by incubation with erythrocyte lysis solution. PBMCs were washed twice with PBS-EDTA, resuspended in 10 mL normal saline and transfused into recipient lambs.

PBMCs collected from 450 mL of blood from donor sheep 4125 (Table 1) were further processed to enrich for B lymphocytes using a magnetic cell sorting system (MACS; Miltenyi Biotech, Auburn, CA). Briefly, PBMCs were resuspended in Hanks-balanced salt solution (HBSS; Invitrogen, Carlsbad, CA) containing 2 mM EDTA and 2% fetal bovine serum (FBS) and divided into three tubes. Each tube was then incubated with either anti-CD72 (2-104, IgM, kindly provided by Dr. Young), anti-CD21 (BAQ15A, IgM; VMRD Inc., Pullman WA) or anti-CD18 (HUH82A, IgG2a; VMRD) murine monoclonal antibodies (mAbs) for 30 min on ice. Cells in each tube were washed twice in HBSS and then incubated with rat anti-mouse IgM- or IgG2a-b- coupled magnetic microbeads (Miltenyi) for 15 min on ice. To determine the enrichment purity by flow cytometry, mAb-labeled cells were incubated with either FITC-labeled goat anti-mouse IgM or IgG2a for 5 min. CD72^+ ^and CD21^+ ^labeled B lymphocytes were separated from other PBMCs by passing through LS MACS columns (Miltenyi), washed, and resuspended in 10 mL normal saline. To assess whether magnetic labeling of cells interferes with cell circulation and prion development, PBMCs were labeled with anti-CD18, anti-IgG2a-b and FITC-IgG2a mAbs. These CD18-labeled PBMCs were not subjected to MACS columns enrichment and were used as a positive control for the study. Approximately 5 × 10^7 ^CD18-labeled PBMCs, 2 × 10^7 ^CD72^+ ^B cells and 7 × 10^6 ^CD21^+ ^B cells were isolated from the initial 50 mL of whole blood and transfused into lambs. Lambs (three animals per group) received CD72^+ ^B cells, CD21^+ ^B cells or CD18-labeled PBMCs. Whole blood, platelet and plasma recipients were held in the quarantine facility in pens with no access to other infected sheep or goats.

### Scrapie diagnosis by immunohistochemistry and western blot assays

Antemortem diagnosis of scrapie was made by biopsy of the rectal mucosa and detection of PrP^Sc ^by IHC. Samples were collected from each recipient lamb as follows: MVRQ/MVRQ recipients: first biopsy at four months and monthly thereafter; MARQ/MVRQ or TARQ/MVRQ recipients: first biopsy at six months and one to two months thereafter; MARQ/MARQ or MARQ/TARQ recipients: first biopsy at eight months and nine, 15, 18, 25 and 36 months thereafter. Animals were euthanized by intravenous administration of a pentobarbital-based euthanasia solution (Vortech, Dearborn, MI) when most of the recipients in the same group became rectal biopsy positive for PrP^Sc ^(Table 1). Antemortem and postmortem rectal tissues, postmortem lymphoid tissues (retropharyngeal lymph nodes, palatine tonsils, spleen, ileo-cecal junction, ileo-cecal lymph nodes and mesenteric lymph nodes) and brains were fixed in formalin and processed according to standard procedures. Three μm sections mounted on treated glass slides (Superfrost^®^/Plus, Fisher Scientific, Pittsburg, PA) were immunolabeled with a combination of mAbs F89/160.1.5 [31] and F99/97.6.1 [5] using an automated immunolabeler (Benchmark, Ventana Medical Systems, Tucson, AZ), and counterstained with hematoxylin as previously described [32]. Positive and negative ovine lymphoid and brain tissues were used as run control samples. Immunolabeling intensity of the positive control tissue was equivalent for all runs and no labeling was observed in negative control tissues or in positive control tissues for which an isotype-matched mAb was substituted for the anti-PrP mAbs. Samples were considered positive for PrP^Sc ^if coarse dark red deposits were detected in the lymphoid follicles or in the dorsal motor nucleus of the vagus nerve at the level of obex by using bright-field microscopy. Photomicrographs were taken with an Olympus BX40 microscope coupled with an Olympus Q-Color3 camera. Axiovision software was used for scaling and Adobe Photoshop Elements 5.0 for formatting. Brain tissue samples from two preclinical and all four clinical donor sheep collected at necropsy were selected for further analysis for PrP^Sc ^by western blot assays as described previously [32-34]. Briefly, proteinase K (50 μg/mL final concentration) was directly added into 100 μl 10% (w/v) brain homogenates and incubated at 50°C for 1 h. Ten microliters of homogenates were mixed with SDS-PAGE sample loading buffer and loaded onto a 12% Nu-PAGE Bis-Tris gel (Invitrogen). After electrophoresis, proteins were transferred onto PVDF membranes, blocked with commercial casein blocker (Pierce, Rockford, IL) and incubated with primary mAb F99/97.6.1 followed by incubation with a horseradish peroxidase conjugated goat anti-mouse secondary Ab (SouthernBiotech, Birmingham, AL). Bound antibody was detected by chemiluminescence (Amersham ECL™, GE healthcare, Piscataway, NJ). Membranes were exposed to radiographic films (KodakBioMax Chemiluminescence Films) and evaluated for di-, mono- and un-glycosylated PrP^Sc ^banding patterns. Positive and negative sheep brain homogenates and isotype-matched mAbs were used as controls for the assay.

## Authors' contributions

KO and RD designed the experiments and analyzed the experimental data. RD prepared the manuscript. KO supervised the experiments and helped draft manuscript. DS performed the post mortem analysis and helped draft the manuscript. AY provide anti-CD72 mAb and helped draft manuscript. TT prepared the tissues, developed sections for immunolabeling and performed all the immunohistochemistry assays. DZ performed the western blot assays. All authors have read and approved the final manuscript.

## References

[B1] Bolton DC McKinley MP Prusiner SB Identification of a protein that purifies with the scrapie prion Science 1982 218 4579 1309 1311 10.1126/science.6815801 6815801

[B2] Prusiner SB Novel proteinaceous infectious particles cause scrapie Science 1982 216 4542 136 144 10.1126/science.6801762 6801762

[B3] van Keulen LJ Schreuder BE Meloen RH Mooij-Harkes G Vromans ME Langeveld JP Immunohistochemical detection of prion protein in lymphoid tissues of sheep with natural scrapie J Clin Microbiol 1996 34 5 1228 1231 8727908 10.1128/jcm.34.5.1228-1231.1996 PMC228987

[B4] O'Rourke KI Baszler TV Parish SM Knowles DP Preclinical detection of PrPSc in nictitating membrane lymphoid tissue of sheep Vet Rec 1998 142 18 489 491 10.1136/vr.142.18.489 9612916

[B5] O'Rourke KI Baszler TV Besser TE Miller JM Cutlip RC Wells GA Ryder SJ Parish SM Hamir AN Cockett NE Preclinical diagnosis of scrapie by immunohistochemistry of third eyelid lymphoid tissue J Clin Microbiol 2000 38 9 3254 3259 10970367 10.1128/jcm.38.9.3254-3259.2000 PMC87369

[B6] Gonzalez L Jeffrey M Siso S Martin S Bellworthy SJ Stack MJ Chaplin MJ Davis L Dagleish MP Reid HW Diagnosis of preclinical scrapie in samples of rectal mucosa Vet Rec 2005 156 26 846 847 15980141 10.1136/vr.156.26.846-b

[B7] Espenes A Press CM Landsverk T Tranulis MA Aleksandersen M Gunnes G Benestad SL Fuglestveit R Ulvund MJ Detection of PrP(Sc) in rectal biopsy and necropsy samples from sheep with experimental scrapie J Comp Pathol 2006 134 2-3 115 125 10.1016/j.jcpa.2005.08.001 16466737

[B8] Houston F Foster JD Chong A Hunter N Bostock CJ Transmission of BSE by blood transfusion in sheep Lancet 2000 356 9234 999 1000 10.1016/S0140-6736(00)02719-7 11041403

[B9] Houston F McCutcheon S Goldmann W Chong A Foster J Siso S Gonzalez L Jeffrey M Hunter N Prion diseases are efficiently transmitted by blood transfusion in sheep Blood 2008 112 12 4739 4745 10.1182/blood-2008-04-152520 18647958

[B10] Hunter N Foster J Chong A McCutcheon S Parnham D Eaton S MacKenzie C Houston F Transmission of prion diseases by blood transfusion J Gen Virol 2002 83 Pt 11 2897 2905 12388826 10.1099/0022-1317-83-11-2897

[B11] McCutcheon S Alejo Blanco AR Houston EF de Wolf C Tan BC Smith A Groschup MH Hunter N Hornsey VS MacGregor IR All clinically-relevant blood components transmit prion disease following a single blood transfusion: a sheep model of vCJD PLoS One 2011 6 8 e23169 10.1371/journal.pone.0023169 21858015 PMC3157369

[B12] Thorne L Terry LA In vitro amplification of PrPSc derived from the brain and blood of sheep infected with scrapie J Gen Virol 2008 89 Pt 12 3177 3184 19008409 10.1099/vir.0.2008/004226-0

[B13] Terry LA Howells L Hawthorn J Edwards JC Moore SJ Bellworthy SJ Simmons H Lizano S Estey L Leathers V Detection of PrPsc in blood from sheep infected with the scrapie and bovine spongiform encephalopathy agents J Virol 2009 83 23 12552 12558 10.1128/JVI.00311-09 19740979 PMC2786716

[B14] Edwards JC Moore SJ Hawthorn JA Neale MH Terry LA PrP(Sc) is associated with B cells in the blood of scrapie-infected sheep Virology 2010 405 1 110 119 10.1016/j.virol.2010.05.023 20646730

[B15] Rubenstein R Chang B Gray P Piltch M Bulgin MS Sorensen-Melson S Miller MW A novel method for preclinical detection of PrPSc in blood J Gen Virol 2010 91 Pt 7 1883 1892 20357038 10.1099/vir.0.020164-0

[B16] Sowemimo-Coker S Kascsak R Kim A Andrade F Pesci S Meeker C Carp R Brown P Removal of exogenous (spiked) and endogenous prion infectivity from red cells with a new prototype of leukoreduction filter Transfusion 2005 45 12 1839 1844 10.1111/j.1537-2995.2005.00640.x 16371036

[B17] Cervia JS Sowemimo-Coker SO Ortolano GA Wilkins K Schaffer J Wortham ST An overview of prion biology and the role of blood filtration in reducing the risk of transfusion-transmitted variant Creutzfeldt-Jakob disease Transfus Med Rev 2006 20 3 190 206 10.1016/j.tmrv.2006.03.007 16787827

[B18] Hewitt PE Llewelyn CA Mackenzie J Will RG Creutzfeldt-Jakob disease and blood transfusion: results of the UK Transfusion Medicine Epidemiological Review study Vox Sang 2006 91 3 221 230 10.1111/j.1423-0410.2006.00833.x 16958834

[B19] Young AJ Dudler L Yamaguchi K Marston W Hein WR Structure and expression of ovine complement receptor type 2 Vet Immunol Immunopathol 1999 72 1-2 67 72 10.1016/S0165-2427(99)00112-9 10614494

[B20] Pan C Baumgarth N Parnes JR CD72-deficient mice reveal nonredundant roles of CD72 in B cell development and activation Immunity 1999 11 4 495 506 10.1016/S1074-7613(00)80124-7 10549631

[B21] Mathiason CK Hayes-Klug J Hays SA Powers J Osborn DA Dahmes SJ Miller KV Warren RJ Mason GL Telling GC B cells and platelets harbor prion infectivity in the blood of deer infected with chronic wasting disease J Virol 2010 84 10 5097 5107 10.1128/JVI.02169-09 20219916 PMC2863796

[B22] Holada K Vostal JG Theisen PW MacAuley C Gregori L Rohwer RG Scrapie infectivity in hamster blood is not associated with platelets J Virol 2002 76 9 4649 4650 10.1128/JVI.76.9.4649-4650.2002 11932431 PMC155088

[B23] Banks WA Niehoff ML Adessi C Soto C Passage of murine scrapie prion protein across the mouse vascular blood-brain barrier Biochem Biophys Res Commun 2004 318 1 125 130 10.1016/j.bbrc.2004.04.009 15110762

[B24] Siso S Jeffrey M Gonzalez L Neuroinvasion in sheep transmissible spongiform encephalopathies: the role of the haematogenous route Neuropathol Appl Neurobiol 2009 35 3 232 246 10.1111/j.1365-2990.2008.00978.x 19473292

[B25] Murayama Y Yoshioka M Okada H Takata M Yokoyama T Mohri S Urinary excretion and blood level of prions in scrapie-infected hamsters J Gen Virol 2007 88 Pt 10 2890 2898 17872544 10.1099/vir.0.82786-0

[B26] Tsukui K Takata M Tadokoro K A potential blood test for transmissible spongiform encephalopathies by detecting carbohydrate-dependent aggregates of PrPres-like proteins in scrapie-Infected hamster plasma Microbiol Immunol 2007 51 12 1221 1231 18094541 10.1111/j.1348-0421.2007.tb04009.x

[B27] Hewitt P vCJD and blood transfusion in the United Kingdom Transfus Clin Biol 2006 13 5 312 316 10.1016/j.tracli.2006.11.006 17188541

[B28] Lefrere JJ Hewitt P From mad cows to sensible blood transfusion: the risk of prion transmission by labile blood components in the United Kingdom and in France Transfusion 2009 49 4 797 812 10.1111/j.1537-2995.2008.02044.x 19170997

[B29] Laegreid WW Clawson ML Heaton MP Green BT O'Rourke KI Knowles DP Scrapie resistance in ARQ sheep J Virol 2008 82 20 10318 10320 10.1128/JVI.00710-08 18632863 PMC2566274

[B30] Baylis M Goldmann W Houston F Cairns D Chong A Ross A Smith A Hunter N McLean AR Scrapie epidemic in a fully PrP-genotyped sheep flock J Gen Virol 2002 83 Pt 11 2907 2914 12388827 10.1099/0022-1317-83-11-2907

[B31] O'Rourke KI Baszler TV Miller JM Spraker TR Sadler-Riggleman I Knowles DP Monoclonal antibody F89/160.1.5 defines a conserved epitope on the ruminant prion protein J Clin Microbiol 1998 36 6 1750 1755 9620413 10.1128/jcm.36.6.1750-1755.1998 PMC104913

[B32] O'Rourke KI Zhuang D Truscott TC Yan H Schneider DA Sparse PrP(Sc) accumulation in the placentas of goats with naturally acquired scrapie BMC Vet Res 2011 7 7 10.1186/1746-6148-7-7 21284878 PMC3041672

[B33] Spraker TR Balachandran A Zhuang D O'Rourke KI Variable patterns of distribution of PrP(CWD) in the obex and cranial lymphoid tissues of Rocky Mountain elk (Cervus elaphus nelsoni) with subclinical chronic wasting disease Vet Rec 2004 155 10 295 302 10.1136/vr.155.10.295 15478500

[B34] Alverson J O'Rourke KI Baszler TV PrPSc accumulation in fetal cotyledons of scrapie-resistant lambs is influenced by fetus location in the uterus J Gen Virol 2006 87 Pt 4 1035 1041 16528055 10.1099/vir.0.81418-0

